# Careworn

**Published:** 1859-02

**Authors:** 


### "CAREWORN"

Is a familiar expression, and conjures at once an image of a face so pale and sad as to show that its owner was utterly disheartened, was weary of himself, of life, and of all the world besides. Many such are met any day in our public streets, feeding upon what is destroying them. It is moral medicine which these unfortunates require; but unhappily the places where the "balm" for sorrow is to be had, free of cost, is not frequented by those who most need its healing power. But calling in at one of these moral "dispensaries" on Fifth Avenue, during the "crisis of '57," we gathered up some prescriptions from the "Doctor" of Divinity which we think ought to be spread broadcast over the whole country as of enduring value; for in cases not a few we have found that it was a diseased mind which was wasting the body into the grave, and no drop or drug, or pill, or bolus known to the apothecary could avail to break up the malady of the heart. And not wishing to assume responsibilities out of our *present* line, we will use the identical words of the great prescriber, leaving it to the reader to compare and find out whether it be according to the law and testimony :

Trials increase with age, but the path of the just shineth more and more unto the perfect day.

Thinking over past trials, in order to rectify them, is most unavailing.

Each trial has its errand—as a bullet its billet. Receive each trial as from God.

Cultivate the habit of regarding daily vexations as trifles.

Never be troubled with trifles, and soon all trouble will appear as trifling.

Daily educate your mind to turn away from trials.

We can't lessen our trials by thinking on them.

You can't mend them by brooding over them.

Your motto should be—" Look forward and go forward."

Let past troubles go, except for thanks or penitence.

Nothing so kills fretfulness as advancing in duty.

Meet a fire with a new fire; meet one engrossing trouble by zeal in some important duty or enterprise.

Many hearts may even now be fretting about yesterday's trials, or to-morrow's engagements.

Don't dwell too much on seeking for consolation. Blessed are they which " endure."

The more disinterested, the more happy will you be. Throw more of self overboard in a storm, and the lighter will the vessel be left.

Trouble not about want of success in worldly business, or that wealth is endangered, or is departing, or is gone.

Aim to reap benefit from your trials.

All unnecessary care tends to evil.

Heaven is perfect freedom from care; Hell is complete vexation.

Examine how we have fallen into a fretful temper.

The cure of fretful care is in religion.

Reflective brooding makes our cares greater.

To nurse our cares is to create more of them.

Trouble comes like a thunderbolt sometimes in a family; and thus are irreligious men daily now driven over the brink of drunkenness, insanity, and suicide.

We don't know how much material wealth has been consumed in the late commercial disasters; but the wear and tear of anxiety, and the shortening of life, must be computed by hundreds of millions.

When trials come without our own fault, it is wrong to brood over them and to fret.

